# Characterization of antimicrobial use and co-infections among hospitalized patients with COVID-19: a prospective observational cohort study

**DOI:** 10.1007/s15010-022-01796-w

**Published:** 2022-04-14

**Authors:** Tilman Lingscheid, Lena J. Lippert, David Hillus, Tassilo Kruis, Charlotte Thibeault, Elisa T. Helbig, Pinkus Tober-Lau, Frieder Pfäfflin, Holger Müller-Redetzky, Martin Witzenrath, Thomas Zoller, Alexander Uhrig, Bastian Opitz, Norbert Suttorp, Tobias S. Kramer, Leif E. Sander, Miriam S. Stegemann, Florian Kurth

**Affiliations:** 1 Charité–Universitätsmedizin Berlin, Corporate Member of Freie Universität Berlin and Humboldt-Universität zu Berlin, Berlin, Germany; 2 Department of Infectious Diseases and Respiratory Medicine, Charité - Universitätsmedizin Berlin, Augustenburger Platz 1, 13353 Berlin, Germany; 3 Labor Berlin Charité Vivantes GmbH, Mikrobiologie and Hygiene, Sylter Str. 2, 13353 Berlin, Germany; 4 German Center for Lung Research (DZL), Berlin, Germany; 5 Institute for Hygiene and Environmental Medicine, Charité - Universitätsmedizin Berlin, Hindenburgdamm 27, 12203 Berlin, Germany; 6 Department of Tropical Medicine, Bernhard Nocht Institute for Tropical Medicine, Bernhard-Nocht-Straße 74, 20359 Hamburg, Germany

**Keywords:** SARS-CoV-2, Antimicrobial stewardship, Antimicrobial resistance, Bloodstream infections, Co-infection

## Abstract

**Purpose:**

To investigate antimicrobial use and primary and nosocomial infections in hospitalized COVID-19 patients to provide data for guidance of antimicrobial therapy.

**Methods:**

Prospective observational cohort study conducted at Charité–Universitätsmedizin Berlin, including patients hospitalized with SARS-CoV-2-infection between March and November 2020.

**Results:**

309 patients were included, 231 directly admitted and 78 transferred from other centres. Antimicrobial therapy was initiated in 62/231 (26.8%) of directly admitted and in 44/78 (56.4%) of transferred patients. The rate of microbiologically confirmed primary co-infections was 4.8% (11/231). Although elevated in most COVID-19 patients, C-reactive protein and procalcitonin levels were higher in patients with primary co-infections than in those without (median CRP 110 mg/l, IQR 51–222 vs. 36, IQR 11–101, respectively; *p* < 0.0001). Nosocomial bloodstream and respiratory infections occurred in 47/309 (15.2%) and 91/309 (29.4%) of patients, respectively, and were associated with need for invasive mechanical ventilation (OR 45.6 95%CI 13.7–151.8 and 104.6 95%CI 41.5–263.5, respectively), extracorporeal membrane oxygenation (OR 14.3 95%CI 6.5–31.5 and 16.5 95%CI 6.5–41.6, respectively), and haemodialysis (OR 31.4 95%CI 13.9–71.2 and OR 22.3 95%CI 11.2–44.2, respectively). The event of any nosocomial infection was significantly associated with in-hospital death (33/99 (33.3%) with nosocomial infection vs. 23/210 (10.9%) without, OR 4.1 95%CI 2.2–7.3).

**Conclusions:**

Primary co-infections are rare, yet antimicrobial use was frequent, mostly based on clinical worsening and elevated inflammation markers without clear evidence for co-infection. More reliable diagnostic prospects may help to reduce overtreatment. Rates of nosocomial infections are substantial in severely ill patients on organ support and associated with worse patient outcome.

**Supplementary Information:**

The online version contains supplementary material available at 10.1007/s15010-022-01796-w.

## Introduction

The Coronavirus Disease 2019 (COVID-19) pandemic, caused by severe acute respiratory syndrome coronavirus-2 (SARS-CoV-2), has major implications for health care worldwide. Bacterial co-infections are common in viral pulmonary infections, such as influenza, significantly contributing to mortality [1, 2]. While co-infections are reportedly low among COVID-19 patients with rates of primary bacterial co-infections of 1–4% [3–6], use of antimicrobial therapy upon admission is reported in up to 74% [7]. The rate of nosocomial co-infections varies between 4 and 50%, depending on disease severity and level of care (non-intensive care unit (non-ICU) vs. ICU) [8]. Data on microbial workup in COVID-19 are sparse with only few studies reporting the most common pathogens or antimicrobial use, and there is hardly any data differentiating between primary and nosocomial co-infections [8, 9]. There is clear research need to define the exact incidence of co-infections at the different phases of COVID-19, antimicrobial susceptibility profiles and risk factors to establish evidence-based antibiotic stewardship interventions for COVID-19 patients.

We performed a prospective observational cohort study investigating antimicrobial use and primary and nosocomial bacterial and fungal pathogens in respiratory and blood samples from hospitalized COVID-19 patients between March and November 2020 in a German tertiary care centre. We analysed patient characteristics, microbiological, clinical and laboratory data, aiming to identify determinants for co-infections, provide evidence for guidance of optimized antimicrobial therapy, and prioritize antimicrobial stewardship interventions.

## Methods

Data collection was performed within the Pa-COVID-19 study, a prospective observational cohort study conducted at a tertiary care university hospital of Charité–Universitätsmedizin Berlin, as described before [10]. The study was approved by the ethics committee of Charité–Universitätsmedizin Berlin (EA2/066/20), conducted according to the Declaration of Helsinki and Good Clinical Practice principles (ICH 1996), and registered in the German and WHO international clinical trials registry (DRKS00021688).

All adult patients with PCR-confirmed SARS-CoV-2 infection admitted between March and November 2020 were eligible for inclusion. Diagnosis and treatment followed national guidelines and was independent of study participation. All samples were taken within standard of care, and all results of microbiological specimen from blood cultures (BCs) and lower respiratory tract samples (RSs) and antimicrobial prescription data are included in this analysis. Comorbidities were assessed using Charlson Comorbidity Index (CCI) [11]. COVID-19 severity was stratified by WHO ordinal scale of clinical improvement [12].

The primary objective was characterizing antimicrobial use and microbiologically confirmed blood stream (BSI) and respiratory co-infections in hospitalized patients with COVID-19, stratified by time of occurrence (≤ 48 h after admission: primary co-infections;  > 48 h after admission: nosocomial co-infections). For primary co-infections, only primarily admitted patients (directly admitted to Charité hospital or transferred ≤ 48 h after admission) were considered.

BSIs were classified as follows: in primary co-infections, isolates of typical skin commensal and coagulase-negative staphylococci (ConS) were excluded due to the high probability of contamination among patients just admitted to the hospital. In nosocomial BSIs, ConS were documented but not included in the analysis of risk factors and outcome because of uncertainty regarding true infection or contamination. For RSs, all isolated pathogens except *Candida* spp. and non-specified yeast were considered possible infectious agents. Among patients on invasive mechanical ventilation (IMV), RS were collected via tracheal aspiration (either via conventional aspiration or bronchoscopy); patients not on IMV who reported productive cough were instructed on submitting a (morning) sputum sample.

Differentiation between primary respiratory co-infection and colonization was made by treating physicians with review by an infectious disease specialist (based on radiological findings, laboratory parameters, and clinical worsening without antimicrobial therapy). Antimicrobial treatment without clear focus mentioned in medical charts was defined “empirical for suspected co-infection”. Courses of C-reactive protein (CRP) and procalcitonin (PCT) were recorded over the hospital stay, and values at the day of onset of every BSI or respiratory tract infection (± 2d) compared to the lowest corresponding value during the 7 days prior.

Laboratory and microbiological analyses were performed at Labor Berlin–Charité Vivantes GmbH, subsidiary company of Charité–Universitätsmedizin, accredited by Deutsche Akkreditierungsstelle (national Accreditation Body of the Federal Republic of Germany). Following patterns were analysed regarding antimicrobial resistance (AMR): carbapenem-resistant (CR) or third-generation cephalosporine-resistant (3GCR) Enterobacterales; CR Pseudomonas (P.) aeruginosa with MDR-phenotype (resistant to ureidopenicillins, cephalosporines and fluoroquinolones or harboring a carbapenemase as defined by national guidelines (1), CR Acinetobacter baumannii-complex; methicillin-resistant Staphylococcus aureus (MRSA), vancomycin-resistant enterococci (VRE).

Regarding microbiological workup, blood culture bottles were incubated in the BACTEC FX blood culture system (BD, Switzerland). Positive bottles were subcultured on conventional solid media and incubated in aerobic and anaerobic atmospheres at 37 °C. Respiratory samples were cultured on conventional solid media and incubated under aerobic conditions. Aerobic microorganisms (bacteria and yeast) were routinely identified using Matrix-Assisted Laser Desorption/Ionization Time-of-Flight Mass Spectrometry (MALDI-TOF) using the VitekMS system or by biochemical means using the VITEK 2 system (bioMérieux, France). Antibiotic susceptibility testing (AST) was conducted in the VITEK 2 System. If necessary, additional commercial methods were applied, such as the disk diffusion method, E-tests or broth microdilution. Anaerobes were identified by MALDI-TOF and tested for susceptibility using ATB ANA (bioMérieux, France) or *E* tests. If *Aspergillus* spp. were cultured from respiratory samples, phenotypic identification of the species complex was done by macroscopic features and microscopic examination of cellotape flag preparations mounted in lactophenol cotton blue. AST was conducted according to recommendations of the European Committee on Antimicrobial Susceptibility Testing (EUCAST) and results were interpreted according to EUCAST Breakpoint tables for interpretation of MICs and zone diameters, version 10.0, 2020 [13].

Distribution of continuous variables was summarized by median and interquartile range (IQR). Differences of continuous variables between groups were examined by Mann–Whitney *U* test. Categorical variables were compared using chi-square and Fisher’s exact tests. For all analyses, complete cases were used for the respective evaluation. A *p* value < 0.05 was considered statistically significant. Analyses were conducted with JMP (version 15 pro, SAS Institute Inc., Cary, United States) and GraphPad Prism (version 9, GraphPad Software Inc., San Diego, United States).

## Results

309 patients hospitalized for COVID-19 were included in this study. Of those, 231 (74.8%) patients were primarily admitted to Charité hospital, whereas 78 patients (25.2%) were transferred from another centre. Patient characteristics are summarized in Table 1.

**Table 1:** Patient characteristics, risk factors and laboratory parameters associated with primary co-infection upon admission

	All patients *N* = 309	Primarily admitted patients *N* = 231 (74.8%)	Transferred patients *N* = 78 (25.2%)	Primarily admitted patients with confirmed co-infection 11/231 (4.76%)	Primarily admitted patients without confirmed co-infection 220/231 (95.23%)	*P* value; odds ratio (95%CI)
Age [median, IQR]	59 (49.5–70)	59 (49–70)	61 (51–69.5)	67 (62–69)	58 (48–71)	0.2
Age ≥ 65 years [*n*/*N* (%)]	128/296 (43.2)	85/230 (37)	22/61 (36.1)	7/10 (70)	78/220 (35.5)	**0.041;** **OR 4.25 (1.07–16.9)**
Male sex [*n*/*N* (%)]	209/305 (68.5)	158/230 (68.7)	51/74 (68.9)	9/11 (81.8)	149/220 (67.7)	0.5; OR 2.1 (0.45–10.2)
CCI [median, IQR]	2 (1–4)	2 (1–4)	3 (1–4)	3 (3–6.25)	2 (1–4)	**0.009**
CCI > 3 [*n*/*N* (%)]	143/291 (49.1)	108/226 (47.8)	32/65 (50.8)	9/10 (90)	99/216 (45.8)	**0.0076;** **OR 10.6 (1.32–85.4)**
Cardiovascular disease [*n*/*N* (%)]	160/279 (57.4)	124/218 (56.9)	36/61 (59)	6/9 (66.7)	118/209 (56.5)	0.73; OR 1.54 (0.37–6.33)
Chronic pulmonary disease [*n*/*N* (%)]	54/286 (18.8)	38/223 (17)	16/63 (25.4)	1/9 (11.1)	37/214 (17.3)	1; OR 0.6 (0.07–4.9)
Diabetes mellitus [*n*/*N* (%)]	64/288 (22.2)	45/224 (20)	19/64 (29.7)	3/10 (30)	42/214 (19.6)	0.42; OR 1.75 (0.43–7.1)
Chronic kidney disease [*n*/*N* (%)]	34/287 (11.8)	30/223 (13.5)	4/60 (6.3)	2/8 (25)	28/215 (13)	0.3; OR 2.2 (0.43–11.6)
WHO at admission^a^ [median, IQR]	4 (3–5)	4 (3–5)	5 (4–7)	6 (4–7)	4 (3–4)	**0.0016**
WHO max. [median, IQR]	5 (4–7)	4 (3–7)	7 (5–8)	7 (6–8)	4 (3–7)	**0.0014**
IMV ≤ 48 h after admission^a^ [*n*/*N* (%)]	88/309 (28.5)	43/231 (18.6)	45/78 (57.7)	7/11 (63.6)	36/220 (16.4)	** < 0.0001;** **OR 8.94 (2.49–32.15)**
CRP (max. 48 h) mg/l [median, IQR]	79.5 (29.25–188.7)	66.55 (23.8–147.9)	178.4 (72.1–327.6)	198.7 (116.17–290.75)	62.6 (22.15–134.9)	**0.004**
CRP > 100 mg/l [*n*/*N* (%)]	119/261 (45.6)	82/206 (39.8)	37/55 (67.3)	8/10 (80)	74/196 (37.8)	**0.016;** **OR 6.6 (1.36–31.9)**
CRP > 200 mg/l [*n*/*N* (%)]	62/261 (23.7)	36/206 (17.5)	26/55 (47.3)	5/10 (50)	31/196 (15.8)	**0.016;** **OR 5.3 (1.45–19.5)**
PCT ng/ml [median, IQR]	0.15 (0.08–0.5)	0.11 (0.07–0.27)	0.96 (0.21–4.17)	0.47 (0.22–3.88)	0.11 (0.07–0.25)	**0.0006**
PCT > 0.5 ng/ml [*n*/*N* (%)]	61/248 (24.6)	25/193 (12.9)	36/55 (65.5)	5/10 (50)	20/183 (10.9)	**0.004;** **OR 8.15 (2.16–30.6)**
Leukocytes Gpt/l [median, IQR]	7.26 (5.35–10.63)	6.8 (5.23–9.31)	11.73 (6.97–15.71)	8.73 (4.9–36.3)	6.775 (5.2–9.24)	0.13
Neutrophils Gpt/l [median, IQR]	5.31 (3.51–8.24)	4.85 (3.38–7.33)	8.56 (5.13–12.89)	8.24 (4.41–9.84)	4.76 (3.38–7.3)	0.054
LDH U/l [median, IQR]	385 (302–521.5)	361.5 (291–491)	430 (360–589)	513, (271–613)	357 (291.5–466.5)	0.19
Ferritin µg/l [median, IQR]	834 (395–1670)	797 (348–1547)	1269 (472–2159)	1163 (605,25–2355,5)	797 (334,35–1524)	0.19

All laboratory parameters are the maximum value obtained during the first 48 h after admission to Charité hospital

*IQR* interquartile range, *CI* confidence interval, *CCI* Charlson comorbidity index, *CRP* C-reactive protein, *LDH* lactate dehydrogenase

^a^Admission to Charité hospital

Bold values indicate p < 0.05

### Antimicrobial use upon admission and transferral

**Fig. 1:**
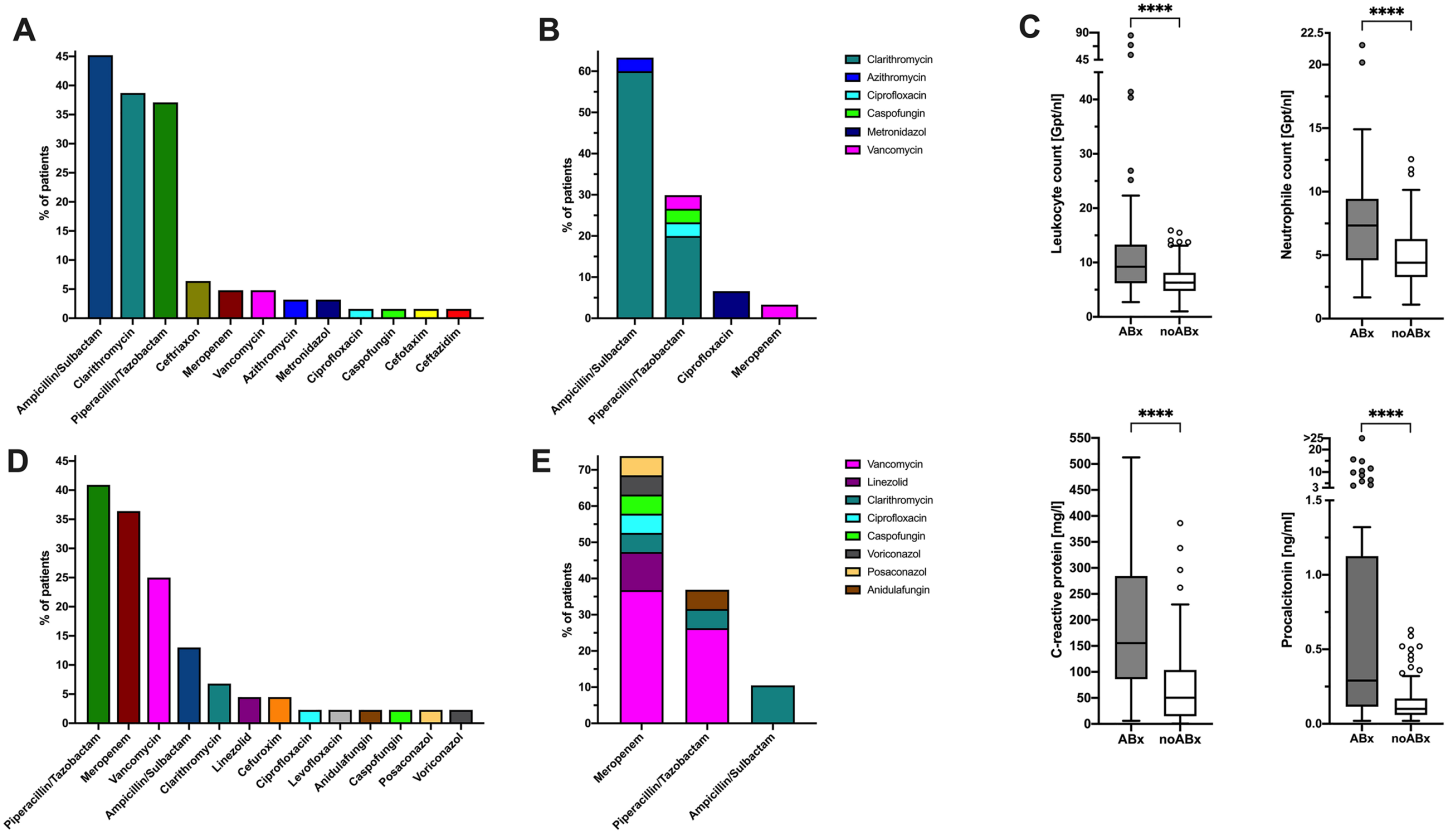
Antimicrobial use at primary admission and after transferral from other centres. **A** Antimicrobial substances and **B** combination therapies given as initial therapy at primary admission, *n* = 62 patients **C** Laboratory parameters of patients who received antimicrobial therapy at primary admission vs. patients who did not (medians): leukocyte count 9.23 (IQR 6.18–13.3) vs. 6.3 (IQR 4.76–8.13) Gpt/nl; neutrophil count 7.34 (IQR 4.6–9.44) vs. 4.41 (IQR 3.27–6.28) Gpt/nl; CRP 155.5 (IQR 68.1–284.38) vs. 50.45 mg/l (IQR 14.73–103.85); PCT 0.29 (IQR 0.12–1.125) vs. 0.1 µg/l (0.06–0.17); all p < 0.0001. **D** Antimicrobial substances and **E)** combination therapies given as initial therapy for transferred patients, *n* = 44 patients. **A**, **B**, **D**, **E** show the percentage of patients receiving the respective antimicrobial, leading to > 100% total because of dual or triple therapies. *ABx* antimicrobial therapy

Among primarily admitted patients, 26.8% (62/231) received antimicrobial therapy within the first 48 h after admission, mainly β-lactam–β-lactamase-inhibitor combinations and macrolides (Fig. 1A). In 48.4% (30/62), combinations of two or more antimicrobials were used (Fig. 1B). The majority (72.6%, 45/62) received empiric treatment for suspected bacterial co-infection without defined infection focus, whereas 17 patients (27.4%) received directed antimicrobial therapy (Supplement Table 1). Initial therapies were discontinued after a median of 4 days (IQR 2–7) and in 24% (15/62) switched to a different substance.

Patients with antimicrobial treatment upon admission had significantly higher levels of leukocytes, neutrophils, CRP, and PCT than those without (Fig. 1C), and were more likely to need invasive mechanical ventilation (IMV) within 48 h (OR 4.39, 95%CI 2.19–8.8, *p* < 0.0001). Age and CCI were similar (median CCI 3, IQR 1–4 vs. 2, IQR 1–3, *p* = 0.19; median age 58 years, IQR 47–71 vs. 60 years, IQR 49–70, *p* = 0.771). Overall, patients receiving antimicrobials upon admission had a more severe disease course, reflected by the maximum score on the WHO ordinal scale during hospital stay (median max. WHO score 6, IQR 4–7 vs. 4, IQR 3–5, in patients with and without antimicrobial treatment, respectively; *p* = 0.0003).

Of the 78 patients transferred to Charité hospital (median duration of prior hospitalisation 7d, IQR 5–13.5), a comparatively higher proportion (56.4%, 44/78) received antibiotic treatment with more broad-spectrum and AMR effective antimicrobials used (Fig. 1D, E).

### Co-infections upon admission

BCs were obtained from 59.3% (137/231, 305 pairs in total) and RSs from 16% (37/231) of patients within 48 h after primary admission. Collection of BCs and RSs were associated with higher levels of CRP and PCT (median CRP 110 mg/l, IQR 50.8–221.5 vs. 35.8, IQR 11.1–100.8, *p* < 0.0001; median PCT 0.2 ng/ml, IQR 0.09–0.63 vs. 0.11 ng/ml, IQR 0.05–0.22, *p* = 0.0004), and higher WHO-score at admission (median 4, IQR 3–5 vs. 3, IQR 3–4, *p* < 0.0001). 37.2% (51/137) of the patients sampled received early antibiotic treatment, and 82.3% (51/62) of the patients treated were sampled.

Six BCs were positive (positivity rate 4.3%, 6/137), three in patients with a defined infection focus (pyelonephritis, cholangitis, perforated peptic ulcer) and three without defined focus. In all three primary BSIs, penicillin-sensitive streptococci were isolated. RSs were positive in 12 patients (positivity rate 32.4%, 12/37), of which four were classified as colonization and eight (3.5%, 8/231) as bacterial superinfection. The isolated pathogens are shown in Supplementary Table 2. Age ≥ 65 years and number of comorbidities were risk factors for primary co-infection in univariate analysis, and patients with co-infections had significantly higher levels of CRP and PCT (Table 1).

### Nosocomial BSIs

**Fig. 2:**
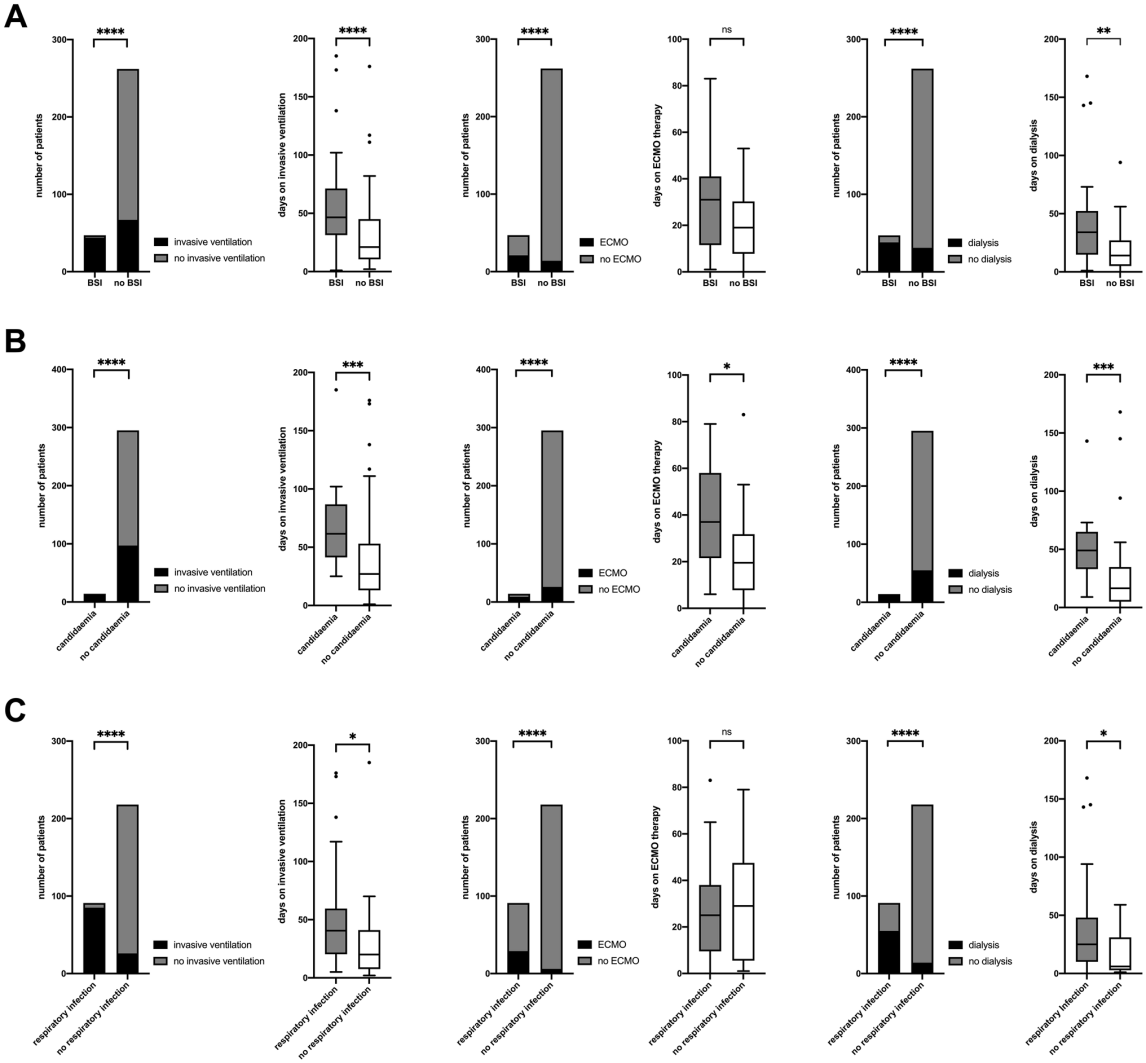
Presence of organ replacement therapies (IMV, ECMO, HD) in patients with and without nosocomial co-infections for **A** patients with and without BSI. On IMV: 93.6%, 44/47 vs. 25.6%, 67/262 (p < 0.0001 OR 45.6 CI 13.7–151.8). IMV duration for a median of 46.5 days (IQR 31.25–71.25) vs. 21 days (IQR 10–45), *p* < 0.0001. On ECMO: 44.7%, 21/47 vs. 5.3%, 14/262 (p < 0.0001, OR 14.3 CI 6.5–31.5). ECMO duration for a median of 31 days (IQR 11.5-41d) vs. 19 days (IQR 7.75–30.25d), *p* = 0.1. On HD: 80.8%, 38/47 vs. 11.8%, 31/262 (p < 0.0001, OR 31.4 CI 13.9–71.2). Duration of HD treatment median of 34 days (IQR 14.75–52.25) vs. 14 days (IQR 5–27), *p* = 0.005. All values are given for patients with and without BSI, respectively **B** Patients with and without Candidemia: on IMV: 100%, 14/14 vs. 32.9% 97/295 (*p* < 0.0001 OR n/a). IMV for a median of 61.5 days (IQR 41.25–86.75) vs. 26 days (IQR 12–52), *p* = 0.0007. On ECMO: 64.3% 9/14 vs. 8.8% 26/295 (*p* < 0.0001, OR 18.6 CI 5.8–59.7). ECMO for a median of 37 days (IQR 21.5–58) vs. 19.5 days (IQR 7.75–31.75), *p* = 0.036. On HD: 100%, 14/14 vs. 18.6%, 55/295 (*p* < 0.0001 OR n/a). Duration of HD median 49 days (IQR 33–65) vs. 16.5 days (IQR 5–34.75), *p* = 0.0003. All values are given for patients with and without candidemia, respectively **C** Patients with and without respiratory co-infection: on IMV: 93.4%, 85/91 vs. 11.9% 26/218 (*p* < 0.0001 OR 104.6 CI 41.5–263.5). IMV for a median of 41 days (IQR 20.25–59.5) vs. 12 days (IQR 6–28), *p* = 0.0001. On ECMO: 31.8%, 29/91 vs. 2.75%, 6/218 (*p* < 0.0001, OR 16.5 CI6.5–41.6). ECMO for a median of 25 days (IQR 9.5–38) vs. 29 days (IQR 5.5–47.5), *p* = 0.94. On HD: 60.4%, 55/91 vs. 6.4%, 14/218 (*p* < 0.0001 OR 22.3 CI 11.2–44.2). HD for a median of 25 days (IQR 10–48) vs. 6 days (IQR 2.75–31), *p* = 0,025. All values are given for patients with and without co-infection, respectively. *IMV* invasive mechanical ventilation; *ECMO* extracorporeal membrane oxygenation; *HD* haemodialysis; *BSI* blood stream infection; *OR* odd’s ratio; *IQR* interquartile range; *C* confidence interval

Nosocomial BSIs > 48 h after primary admission were found in 47/309 patients (15.2%). Median time from admission to first positive BC was 20d (IQR 13–27). In 17 patients (5.5%), a second BSI occurred after a median of 48d (IQR 29.7–78), seven patients (2.2%) experienced ≥ 3 BSIs. In total, 92 bacterial or fungal isolates were detected in 80 BCs (Table 2). ConS were isolated from 67 BCs which were not included in the further analysis as stated above. All BSIs occurred among ICU patients (47/47 (100%), *p* < 0.0001) and were associated with need (OR 42.7, 95%CI 12.8–142) and longer duration of IMV, as well as need (OR 31.4, 95%CI 13.9–71.2) and longer duration of haemodialysis (HD). The need for extracorporeal membrane oxygenation therapy (ECMO) (OR 14.3, 95%CI 6.5–31.5) but not duration of ECMO was associated with the occurrence of BSIs (Fig. 2A).

**Table 2:** Bloodstream and respiratory isolates

Bloodstream isolates	Total number (*N* = 159)	AMR 17/159 (10.6%)	Median days from admission to isolation (IQR)
Coagulase negative Staphylococci	67	–	18 (10.75–37.75)
*Enterococcus* spp.	27		
*Enterococcus faecium*	22	4 (18.1%)^c^	48 (21–91)
*Enterococcus faecalis*	5	–	32 (9–42)
*Candida* spp.	15	–	23.5 (16–43.5)
*Candida glabrata*	8	–	
*Candia albicans*	3	–	
*Candida parapsilosis*	3	–	
*Candida dubliniensis*	1	–	
*Escherichia coli*	8	1 (12.5%)^d^	38 (18.5–63.25)
*Acinetobacter baumannii-complex*	8	8 (100%)^e^	23.5 (13.25–44.75)
*Pseudomonas aeruginosa*	8	3 (37.5%)^f^	20.5 (8.5–81.5)
*Klebsiella* spp.	8		23 (13.75–31.25)
*Klebsiella pneumoniae*	5	1 (20%)^g^	
*Klebsiella aerogenes*	1	–	
*Klebsiella oxytoca*	1	–	
*Klebsiella variicola*	1	–	
*Streptococcus mitis/oralis*	1	–	
*Streptococcus anginosus*	1	–	
*Serratia marcescens*	3	–	
*Morganella morganii*	2	–	
*Staphylococcus aureus*	2	–	
Diverse others*	9	–	

Respiratory isolates	Total number (*N* = 206)	AMR 32/206 (15.5%)	Median days from admission to isolation (IQR)
*Klebsiella* spp.	46		13 (8.75–21)
*Klebsiella pneumoniae*	32	6 (18.8%)^h^	13.5 (10–24.75)
*Klebsiella aerogenes*	10		11 (5–18)
*Klebsiella oxytoca*	4		5 (4–10.5)
*Escherichia coli*	28	5 (17.9%)^i^	18.5 (10–28.5)
*Pseudomonas aeruginosa*	24	3 (12.5%)^j^	22 (17.25–39)
*Staphylococcus aureus*	22		6 (4–14.25)
*Achromobacter spp*	14		24.5 (18.5–33.25)
*Aspergillus spp*	15		19 days (IQR 4–37)
*Citrobacter koseri*	10	4 (40%)^k^	27 (7.25–40.25)
*Proteus mirabilis*	8		18 (13.5–19)
*Acinetobacter baumannii-complex*	7	7 (100%)^l^	13 (6–19)
*Enterobacter cloacae*	6	3 (50%)^m^	22 (15–29.5)
*Citrobacter freundii*	5	3 (60%)^n^	35 (9.5–42.5)
*Serratia marcescens*	4	1 (25%)^o^	21.5 (11–110)
*Haemophilus influenzae*	2		
*Morganella morganii*	2		
*Stenotrophomonas maltophilia*	2		
*Streptococcus agalactiae*	2		
*Streptococcus pneumoniae*	2		
Others **	7		

*3 × Anaerobes, A*rthrobacter crystallopoietes, Chryseobacterium indologenes, Comamonas testosteroni, Enterobacter cloacae, Proteus mirabilis,*

*Raoultella ornithinolytica*: included bacteria, where clear distinction between relevant BSI and contamination was not possible

**Streptococcus constellatus, Acinetobacter lwoffii, Chryseobacterium indologenes, Raoultella planticola, 3 × *Pseudomonas* spp.

AMR: antimicrobial resistance, CR: carbapenem-resistant, 3GCR: third-generation cephalosporine-resistant, CR: carbapenem-resistant, VRE: vancomycin-resistant enterococci

^c^4/4 VRE

^d^1/1 3GCR *E. coli*

^e^8/8 CR *A. baumannii*

^f^3/3 CR *P. aeruginosa*

^g^1/1 3GCR *K. pneumonia*

^h^5/6 3GCR, 1/6 CR

^i^5/5 3GCR

^j^3/3 CR

^k^4/4 3GCR

^l^7/7 CR

^m^3/3 3GCR

^n^3/3 3GCR

^o^1/1 3GCR

The event of a BSI during hospital stay was associated with in-hospital death (23/47, 48.9% case fatality in patients with BSI vs. 33/262, 12.6%, *p* < 0.0001, OR 6.7, 95%CI 3.4–13). Use of dexamethasone, in standard dose recommended for COVID-19 [13], was not associated with the occurrence of BSIs (19/47, 40.4% of patients with BSI received dexamethasone vs. 75/230 32.6% of patients without BSI; *p* = 0.3 OR 1.4 95%CI 0.74–2.68, data missing for 32 patients) in this cohort but was significantly associated with occurrence of more than one BSI (10/17, 58.8% of patients with > 1 BSI had dexamethasone vs. 84/260, 32.3% of patients with < 1 BSI *p* = 0.025, OR 3 95%CI 1.1–8.14).

Fifteen BSIs in 14 patients were caused by *Candida* spp. (Table 2). All patients were severely ill (WHO scale ≥ 7), the association of candidaemia with IMV, HD and ECMO are shown in Fig. 2B. Occurrence of candidaemia was associated with longer hospitalization (65.5d, IQR 44.25–101.5, vs. 14d, IQR 8–32, *p* < 0.0001) and higher mortality (7/14, 50% vs. 49/295, 16.6% p = 0.0015, OR 5 95%CI 1.8–13.5). Use of dexamethasone was not associated with occurrence of candidaemia (5/14, 35.7% dexamethasone in patients with candidaemia vs. 89/263, 33.8% dexamethasone in patients without, *p* = 0.88, OR 1.09 95%CI 0.35–3.3).

### Nosocomial respiratory co-infections

Nosocomial microbiologically confirmed bacterial respiratory co-infections occurred in 91 patients (91/309, 29.4%). First positive samples were obtained in median 13d (IQR 6–21) after admission. 44 patients (44/309, 14.2% of all; 44/91, 48.4.% of those with first sample) had a second positive sample in median 19d (IQR 15–29) after admission, and 20 patients (20/309, 6.5% of all; 20/91, 22% of those with first sample) had more than 2 positive samples. In total, 163 nosocomial respiratory infections with 206 isolates were recorded (Table 2).

Respiratory co-infections occurred mainly in patients on IMV (85/91, 93.4% vs. 26/218, 11.9%, OR 104.6 95%CI 41.5–263.5 *p* < 0.0001), where the first positive RS was obtained after a median of 11d (IQR 5–19) after intubation and the second sample after 18.5d (IQR 12–28.5), and were further associated with ECMO and HD (Fig. 2C). In-hospital-mortality in patients with nosocomial respiratory co-infection was 30.7% (28/91) compared to 13.2% in those without (28/213) (*p* = 0.0003, OR 2.9 95%CI 1.6–5.3). In total, 206 isolates (without relapses or repeated cultures) were found in 163 RSs of 91 patients. The spectrum of isolated pathogens differed depending on the duration of illness. Gram-positive bacteria comprised 16.6% (20/120) of the first RS, but only 7% (4/57) and 9% (2/22) of the second and third positive samples, respectively. Median time from admission to isolation of gram-positive bacteria was 6d (IQR 4–15) vs. 19d (IQR 12–31) for gram-negative bacteria (*p* < 0.0001). Changes of microbiological spectrum over time from predominantly gram-positive to gram-negative isolates are illustrated in Supplementary Fig. 1.

*Aspergillus* (*A.*) spp. was isolated in RSs of 16 patients (16/309, 5.1%, 13 *A. fumigatus*, three *A. niger*) plus one positive galactomannan test without cultural growth of *Aspergillus* spp.. All patients with *Aspergillus* spp. isolates were on IMV. Median time from admission and intubation to isolation of *Aspergillus* spp. was 19d (IQR 4–37) and 15d (IQR 3.5–34), respectively. Computed tomography (CT) substantiated the diagnosis of invasive pulmonary aspergillosis (IPA) in two cases, eight patients showed consolidations consistent with possible IPA, and five patients had no radiological signs of IPA (CT scans missing for two patients). Case fatality in patients with detection of *Aspergillus* spp. was 47% (8/17) and not significantly higher than in patients without *Aspergillus* spp. (34%, 31/91; OR 1.7 95%CI 0.6–4.9, *p* = 0.3). Use of dexamethasone was significantly associated with detection of *Aspergillus* spp. (10/17, 58.8% dexamethasone use in patients with *A*. spp. vs. 84/260, 32.3% in patients without, OR 3 95%CI 1.1–8.13, *p* = 0.034).

### AMR and antimicrobial use over hospital stay

Overall, half of the patients (152/309, 49.2%) in the studied cohort received antimicrobial therapy at least once during their hospital stay, 131 of those (86.2%) were treated on ICU at some point. Among those treated, a median of 3.5 different antimicrobials was used (IQR 2–7). AMR pathogens were isolated in 37/309 (12%) patients, all treated on ICU, and patients with detection of AMR pathogens received a median of seven antimicrobials (IQR 4–10). Among BSIs (Table 2), a small proportion of pathogens exhibited AMR, except *A. baumannii* isolates. Among RSs, 15% (18/120) of first isolated pathogens displayed AMR (five CR and 13 3GCR isolates), which increased slightly to 22.8% (13/57) in isolates of the second RS (five CR and eight 3GCR isolates).

### Laboratory markers

**Fig. 3:**
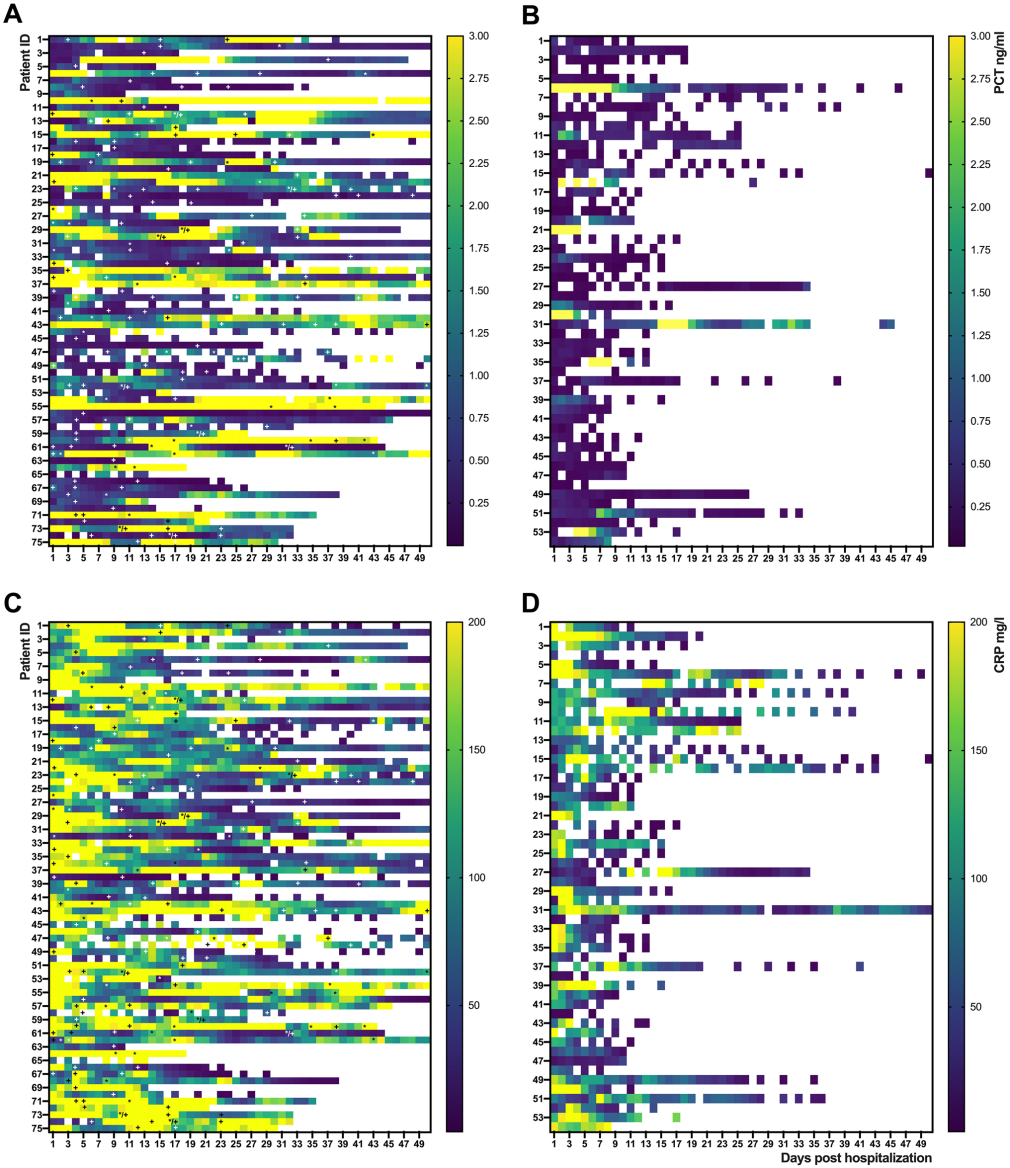
Course of PCT and CRP of COVID-19 patients on ICU with and without nosocomial co-infections. Heat map visualization of PCT and CRP levels over time (d1-50 beginning on admission to Charité hospital) of patients with nosocomial infections (left panels, **A** PCT and **C** CRP) and without proven nosocomial infection (right panels, **B** PCT and **D** CRP). Every row represents one patient and each column 1 day. In **A** and **C** “** + **” represents a positive microbiological respiratory sample and “*” a confirmed blood stream infection. *CRP* c-reactive protein; *PCT* procalcitonin; *d* day

Patients with confirmed co-infections had higher levels and more spikes of CRP and PCT than patients without (Fig. 3A–D). Onsets of BSIs were accompanied with a median PCT increase of 233% (IQR 67.4–854.5%) and a median CRP increase of 162% (IQR 104–439%). Laboratory changes were less pronounced in respiratory infections, with a median PCT increase of 18.9% (-14.1–224.2%) and CRP increase of 114.9% (IQR 14.61–290.5%). There was no difference in the levels of CRP, PCT or leucocyte count regarding the isolation of gram-positive, gram-negative, or fungal pathogens. Supplementary Table 3 summarizes inflammatory parameters in relation to nosocomial infections.

## Discussion

We present in-depth data on antimicrobial use and primary and nosocomial co-infections within a prospective cohort study of hospitalized COVID-19 patients, identifying risk factors for the primary and nosocomial co-infections and providing evidence of significantly worse outcome in co-infected patients with COVID-19. We included ICU and non-ICU patients, and both primarily admitted patients and patients referred due to clinical worsening and need for acute respiratory distress syndrome (ARDS) therapy including ECMO. Thus, this data is representative of the heterogeneous disease course of patients hospitalized for COVID-19 and reflects a real-life clinical picture.

BC sampling upon admission was performed in nearly 60% of patients, while respiratory sampling was performed in only 16%. Sparse respiratory sampling in COVID-19 has been reported previously and might be partly attributable to safety concerns [5]. Sampling was significantly associated with increased CRP und PCT and clinical worsening. Almost half of the patients with RS taken were already on IMV, facilitating sampling also regarding safety concerns of SARS-CoV-2 aerosolization.

Primary microbial co-infections were rare in our cohort (4.8%) and particularly primary BSIs were negligible at around 1%. These findings are slightly lower than previous studies reporting BSI rates of around 3% [5, 14, 15]. Primary pulmonary co-infections were more frequent with 3.5% and comprised mostly gram-positive bacteria, which is in accordance with prior studies [8]. Older age and number of comorbidities were identified as risk factors. Compared with bacterial co-infections in influenza, where they add significantly to morbidity and mortality, incidence of primary bacterial co-infections is drastically lower in COVID-19 [2, 16]. In contrast to the small numbers of primary co-infections, 26.8% of patients received antimicrobial treatment at primary admission. Upfront antimicrobial therapy was associated with and probably triggered by increased inflammatory parameters, disease severity, and clinical deterioration, which is in line with other reports [9]. Thus, high inflammation parameters trigger anti-infective therapy which actually are elevated in case of a primary co-infection, however, initiated antimicrobial therapies outnumber true co-infections by far. About three-fourths of patients were treated empirically, mainly for bacterial pulmonary co-infection analogous to community acquired pneumonia. Herein, the use of macrolides, whose anti-inflammatory role is known for severe pneumonia yet has no proven efficacy in COVID-19, [17, 18] requires further research. That 56.4% received antimicrobial treatment and more broad-spectrum substances at referral is likely reflective of their critical condition and need of ICU-treatment.

Compared to previous studies, we report higher numbers of nosocomial microbial co-infections (15% of all patients), which might be explained by the concise sampling practice and the high proportion of ICU-patients. In addition, we used a longitudinal approach and did not focus on early infections only [3, 19]. Nosocomial infection rate on ICU was 28% in our cohort and is in line with what has been reported before [20]. Respiratory co-infections occurred in 29.4% of all patients, most of them on IMV with an overall rate of 52.7%. The event of any nosocomial bloodstream or respiratory infection was significantly associated with longer hospital stay (44 days (IQR 30–73) vs. 10d (7–15), *p* < 0.0001) and in-hospital death (33.3%, 33/99 vs. 11.2%, 23/205) with an odds ratio of 3.95 (CI95% 2.16–7.22, *p* < 0.0001) reflecting the seriousness of these co-infections.

We found considerable numbers of enterococci, *Candida* spp., *P. aeruginosa* and *Enterobacteriaceae* in nosocomial BSIs*.* The frequency of *A. baumannii* and *P. aeruginosa* isolates with AMR was caused by a contained outbreak on two ICUs. Taking into consideration that all BSIs occurred in ICU patients, most on more than one organ replacement therapy, the pathogen spectrum reflects invasive ICU therapy rather than patient characteristics. In detail, we show that IMV, ECMO and HD are significantly associated with nosocomial infections. In line with this finding, duration of organ replacement therapies were associated with candidaemia, possibly explained by multiple and longer inserted catheters.

Our study has some limitations. It was performed in an ARDS/ECMO referral centre with a patient collective likely to be severely ill. Urinary samples and viral co-infections were not included, and sampling was done as part of clinical standard procedures, so we cannot exclude co-infections missed due to incorrect sampling.

In summary, we show that primary microbial co-infections in COVID-19 are rare. Nosocomial co-infections are associated with severe disease and organ replacement therapies and significantly contribute to COVID-19 mortality. Given the low numbers of pathogens isolated yet high antimicrobial use, antimicrobial therapy in COVID-19 should be accompanied by antimicrobial stewardship interventions. Our study adds solid data on incidence and causative organisms of co-infections in COVID-19, a basis for informed guidance of empirical antimicrobial therapy to optimize patient outcomes and combat the spread of AMR.

## Supplementary Information

Below is the link to the electronic supplementary material.

Supplementary file1 (DOCX 267 KB)
