# Supplementary material for: Characterization of antimicrobial use and co-infections among hospitalized patients with COVID-19: a prospective observational cohort study
Source: Infection. 2022 Apr 14;50(6):1441–52. doi: 10.1007/s15010-022-01796-w (PMC9008382; doi:10.1007/s15010-022-01796-w)
Supplement: Supplementary file 1 — Supplementary file1 (DOCX 267 KB) [file 15010_2022_1796_MOESM1_ESM.docx]

**Supplemental Material**

**Methods:**

**Definition of antimicrobial resistant pathogens**

Following patterns were analysed regarding antimicrobial resistance (AMR): carbapenem-resistant (CR) or third-generation cephalosporine-resistant (3GCR) Enterobacterales; CR *Pseudomonas (P.) aeruginosa* with MDR-phenotype (resistant to ureidopenicillins, cephalosporines and fluoroquinolones or harboring a carbapenemase as defined by national guidelines (1), CR *Acinetobacter baumannii-complex*; methicillin-resistant *Staphylococcus aureus* (MRSA), vancomycin-resistant enterococci (VRE).

**Microbiological Workup:**

Blood culture bottles were incubated in the BACTEC FX blood culture system (BD, Switzerland). Positive bottles were subcultured on conventional solid media and incubated in aerobic and anaerobic atmospheres at 37°C. Respiratory samples were cultured on conventional solid media and incubated under aerobic conditions. Aerobic microorganisms (bacteria and yeast) were routinely identified using Matrix-Assisted Laser Desorption/Ionization Time-of-Flight Mass Spectrometry (MALDI-TOF) using the VitekMS system or by biochemical means using the VITEK 2 system (bioMérieux, France). Antibiotic susceptibility testing (AST) was conducted in the VITEK 2 System. If necessary, additional commercial methods were applied such as the disk diffusion method, E-tests or broth microdilution. Anaerobes were identified by MALDI-TOF and tested for susceptibility using ATB ANA (bioMérieux, France) or E-tests. If *Aspergillus* spp. were cultured from respiratory samples, phenotypic identification of the species complex was done by macroscopic features and microscopic examination of cellotape flag preparations mounted in lactophenol cotton blue. AST was conducted according to recommendations of the European Committee on Antimicrobial Susceptibility Testing (EUCAST) and results were interpreted according to EUCAST Breakpoint tables for interpretation of MICs and zone diameters, version 10.0, 2020 (http://www.eucast.org).

**Supp. Table 1: Indications for directed antimicrobial therapy at admission**

|  | **Indication for directed antimicrobial therapy** | | **Number of patients** |
| --- | --- | --- | --- |
| **Primarily admitted patients or patients transferred within 48h after initial hospitalization** | |  | |
|  | Neutropenia and fever after chemotherapy | | 1 |
|  | Traveller’s diarrhoea (South East Asia) | | 1 |
|  | Cholangitis | | 2 |
|  | Superinfected venous ulcer | | 1^#^ |
|  | Trauma with open fracture | | 2 |
|  | Lobar infiltrate, productive cough, purulent sputum | | 4^#^ |
|  | LVAD, suspected drive-line infection | | 2^*^ |
|  | Urinary tract infection incl. pyelonephritis | | 5^*^ |
|  | Perforated peptic ulcer | | 1 |
|  | #, * each indicating one patient with two indications present | | |
| **Patients transferred after being hospitalized >48h** | | | |
|  | Positive microbiological sample at referring hospital | | 7 |
|  | Urinary tract infection incl. pyelonephritis | | 2 |
|  | Lobar infiltrate, productive cough, purulent sputum | | 1 |
|  | Tracheal rupture with subcutaneous and mediastinal emphysema | | 1 |
|  | Soft tissue abscess | | 1 |

**Supp. Table 2: Isolated bacteria from blood cultures and respiratory samples upon admission or <48h after admission**

| **Isolated bacteria from blood cultures** |
| --- |
| 1 *Streptococcus pneumoniae*  1 *Streptococcus anginosus*  1 *Streptococcus constellatus*  1 *Streptococcus salivarius ^#^*  2 *Escherichia coli* ^#^ |
| **Isolated bacteria from respiratory isolates** |
| 6 *Staphylococcus aureus**  2 *Escherichia coli*  *1 Streptococcus pneumonia*  *1 Klebsiella oxytoca**  *1 Klebsiella pneumoniae*  *1 Haemophilus influenza*  *1 Enterobacter cloacae*  *1 Achromobacter spp*  *1 Bordetella bronchisepticum* |

# BSI, associated other clinical focus (pyelonephritis, cholangitis, perforated peptic ulcer)
* three isolates of *S. aureus* and one isolate of *Klebsiella oxytoca* were classified as colonization based on clinical judgement

**Supp. Table 3: Changes of laboratory values regarding nosocomial infections**

|  | | Median baseline | IQR | Median onset | IQR | Median increase | IQR |
| --- | --- | --- | --- | --- | --- | --- | --- |
| **BSI** | CRP (mg/l) | 70.5 | 35-133 | 250 | 138-331 | 16% | 104-439% |
|  | PCT (ng/ml) | 1.1 | 0.375-2l | 3.5 | 1.46-8 | 233% | 67.4-854.5% |
|  | WBC (Gpt/l) | 8 | 6-11 | 10.4 | 6.35-14.57 | 28.8% | -15.8-70.4% |
| **Respiratory Infection** | CRP (mg/l) | 66.5 | 35-127.75 | 188 | 83.5-308.75 | 114.9% | 14.61-290.5% |
|  | PCT (ng/ml) | 0.44 | 0.2-1.02 | 0.83 | 0.22-283 | 18.9% | -14.1-224.2% |
|  | WBC (Gpt/l) | 8.1 | 6.3-10.14 | 11.5 | 8.25-15.27 | 36.9% | 0-72.6% |

BSI: bloodstream infection, CRP: C-reactive protein, PCT: procalcitonin; WBC: white blood count; IQR: interquartile range

**Supp. Figure 1: Microbiological spectrum in isolates from first and second respiratory samples obtained from patients with COVID-19.**

**
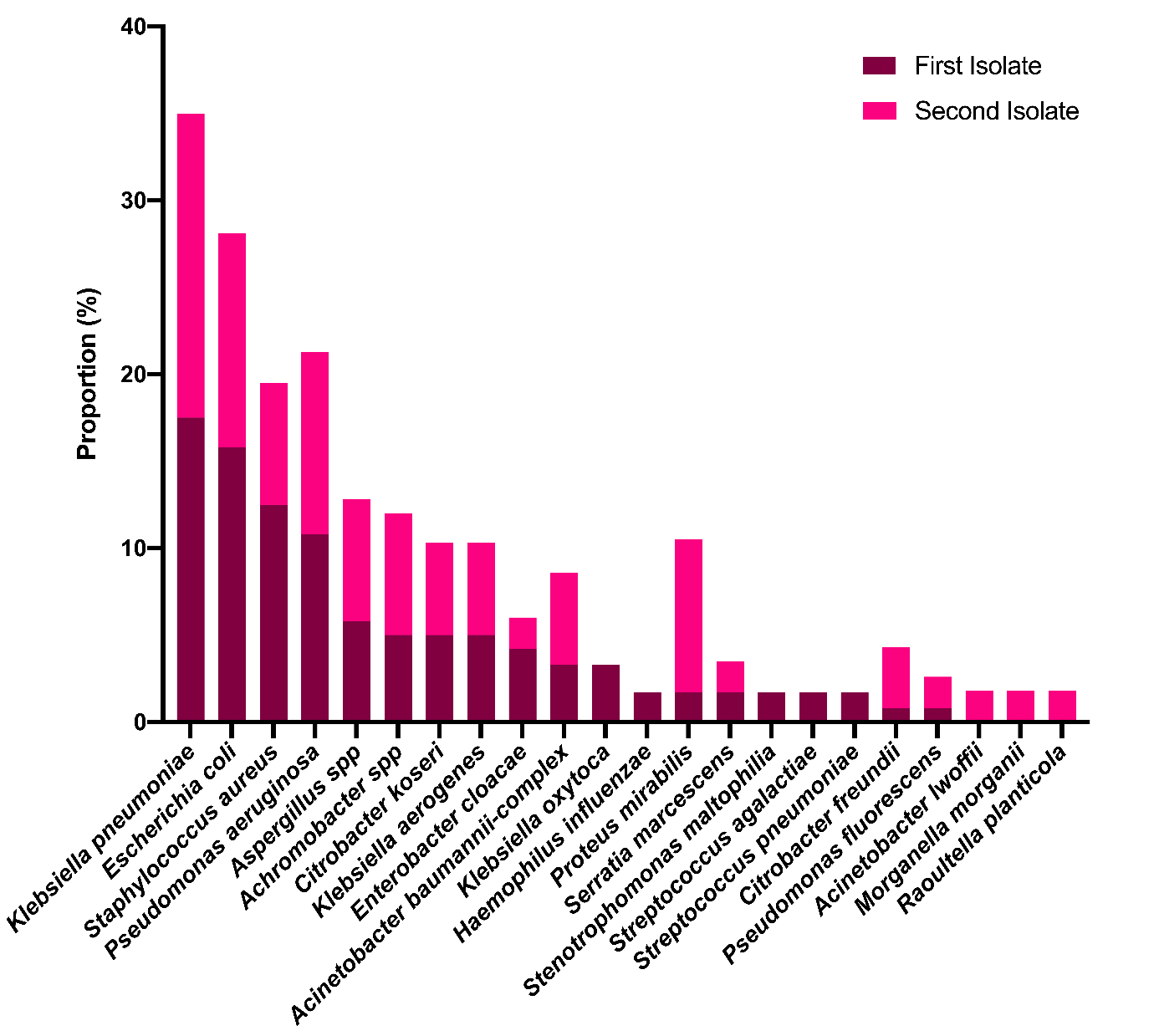
**

First isolate refers to microbiological spectrum among firstly positive respiratory samples (n=91); second isolate refers to patients with a second positive respiratory sample (n=44).

**References:**

1. Hygienemaßnahmen bei Infektionen oder Besiedlung mit multiresistenten gramnegativen Stäbchen. Bundesgesundheitsblatt - Gesundheitsforschung - Gesundheitsschutz. 2012;55(10):1311-54.
